# Polyploidy mitigates the impact of DNA damage while simultaneously bearing its burden

**DOI:** 10.1038/s41420-024-02206-w

**Published:** 2024-10-13

**Authors:** Kazuki Hayashi, Kisara Horisaka, Yoshiyuki Harada, Yuta Ogawa, Takako Yamashita, Taku Kitano, Masahiro Wakita, Takahito Fukusumi, Hidenori Inohara, Eiji Hara, Tomonori Matsumoto

**Affiliations:** 1 Department of Molecular Biology, Research Institute for Microbial Diseases, Osaka University, Osaka, Japan; 2 Department of Otorhinolaryngology-Head and Neck Surgery, Osaka University Graduate School of Medicine, Osaka, Japan; 3 Laboratory of Ploidy Pathology, Graduate School of Frontier Bioscicences, Osaka University, Osaka, Japan; 4 Division of Gastroenterology, Department of Internal Medicine, Kobe University Graduate School of Medicine, Kobe, Japan; 5 Department of Gastrointestinal Surgery, Graduate School of Medicine, Kyoto University, Kyoto, Japan; 6 Laboratory of Aging Biology, Immunology Frontier Research Center, Osaka University, Osaka, Japan

**Keywords:** Senescence, Cancer genetics

## Abstract

Polyploidy is frequently enhanced under pathological conditions, such as tissue injury and cancer in humans. Polyploidization is critically involved in cancer evolution, including cancer initiation and the acquisition of drug resistance. However, the effect of polyploidy on cell fate remains unclear. In this study, we explored the effects of polyploidization on cellular responses to DNA damage and cell cycle progression. Through various comparisons based on ploidy stratifications of cultured cells, we found that polyploidization and the accumulation of genomic DNA damage mutually induce each other, resulting in polyploid cells consistently containing more genomic DNA damage than diploid cells under both physiological and stress conditions. Notably, despite substantial DNA damage, polyploid cells demonstrated a higher tolerance to its impact, exhibiting delayed cell cycle arrest and reduced secretion of inflammatory cytokines associated with DNA damage-induced senescence. Consistently, in mice with ploidy tracing, hepatocytes with high ploidy appeared to potentially persist in the damaged liver, while being susceptible to DNA damage. Polyploidy acts as a reservoir of genomic damage by mitigating the impact of DNA damage, while simultaneously enhancing its accumulation.

## Introduction

Various cell types physiologically become polyploid in humans and polyploidization is closely related to terminal cellular differentiation, as observed in megakaryocytes, cardiomyocytes, and hepatocytes [1]. Although polyploidization has been supposed to have biological importance, such as the enhancement of metabolic activity, transcriptome analyses have shown that gene expression in diploid and polyploid cells under physiological conditions is highly similar on a global scale [2–4]. In addition to physiological conditions, polyploidization is also induced during aging and tissue injury. In the human liver, the ploidy of hepatocytes gradually increases until adolescence and is prominently enhanced in the cirrhotic liver; however, the mechanisms underlying these ploidy alterations are not fully understood [5]. Cancer cells are also frequently polyploid, and pan-cancer analyses have revealed that approximately 38% of human cancers have undergone polyploidization [6]. Although the frequent occurrence of polyploidization in aging, tissue injury, and cancer implies its potential implications in disease pathogenesis, its impact on cellular fate and function remains unclear.

Among the conditions involving dynamic ploidy alterations, the importance of polyploidization in cancer has been relatively well examined [7, 8]. Trajectory analysis of cancer genomes has estimated that a subset of cancers stem from cells that harbor key oncogenic mutations, followed by polyploidization [9, 10]. Moreover, polyploid giant cancer cells (PGCCs), characterized by polyploidy and a markedly large cellular appearance, can be a source of chemotherapy resistance in cancers [11, 12]. Chromosomal instability is thought to be a critical consequence of polyploidy that influences cellular fate and drive cancer evolution [13]. Owing to their excessive number of chromosomes, polyploid cells are prone to chromosome missegregation during mitosis, leading to chromosomal instability, which in turn drives cancer initiation and/or progression by enhancing the heterogeneity of cellular characteristics. Another possible effect of polyploidy is its buffering effect on genetic alterations owing to genome redundancy. Theoretically, multiple alleles in polyploid cells reduce the risk of losing genes that are crucial for cellular survival, leading to enhanced cellular robustness. However, a recent study has shown that polyploidization enhances replication stress during the subsequent cell cycle, leading to the induction of genomic DNA damage shortly after polyploidization [14]. It remains unclear how polyploidy influences the cellular stress burden associated with intrinsic and extrinsic genomic damage.

In this study, we investigated the effect of polyploidization on the cellular response to DNA damage. Polyploidization and accumulation of genomic DNA damage can mutually induce each other, although not necessarily. In addition to cells immediately after polyploidization, stable polyploid cells also sustain an elevated risk of genomic DNA damage compared to diploid cells under both physiological and stress conditions. Notably, despite the prominent accumulation of DNA damage, polyploid cells were more tolerant of its impact, exhibiting delayed cell cycle arrest and a suppressed secretory phenotype associated with DNA damage-induced senescence. Polyploidy mitigates the impact of DNA damage while simultaneously bearing its burden, and is markedly involved in characterizing heterogeneous senescent cells.

## Results

### Cellular senescence induced by the accumulation of DNA damages is often, but not always, accompanied by polyploidy

**Fig. 1:**
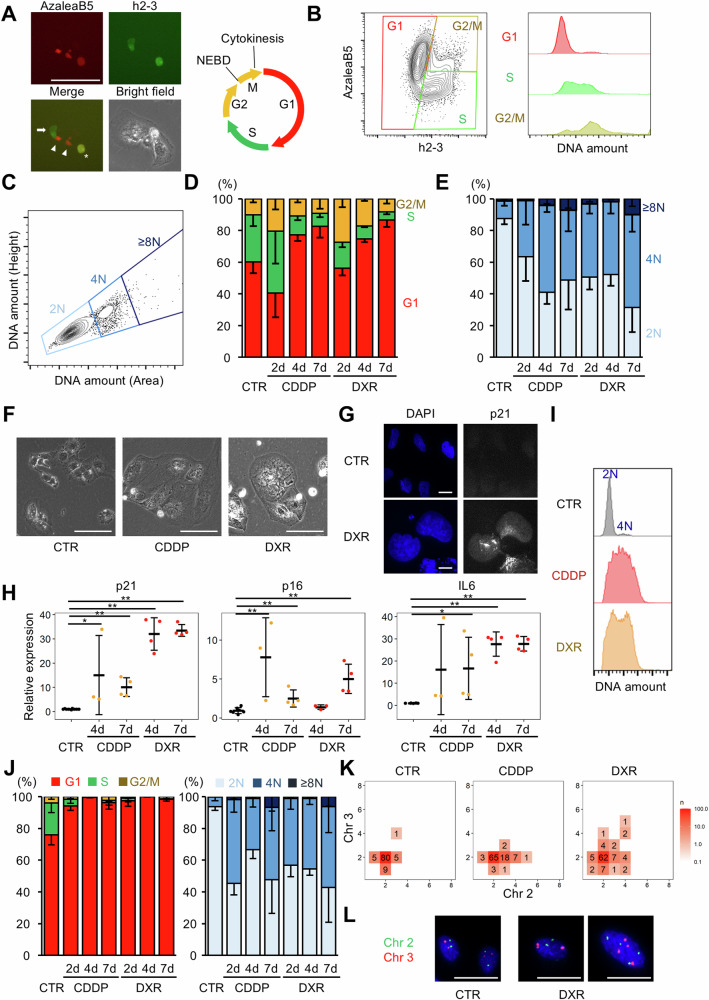
Senescence induction and ploidy alterations induced by DNA-damaging agents. **A** Schematic and fluorescence images of Huh7-Fucci cells. AzaleaB5 (red)-single-positive (arrowheads), h2–3 (green)-single-positive (arrows), and AzaleaB5/h2-3 double-positive (asterisk) cells represent cells in the G1, S, and G2/M phases, respectively. **B**, **C** FACS plot of Huh7-Fucci cells. Only cells in G1 phase are shown in **C**. The detailed gating strategies are presented in Fig. S1. **D** Frequency of Huh7-Fucci cells in G1, S, and G2/M phases. **E** Ploidy distribution of Huh7-Fucci cells in the G1 phase. In **D** and **E**, error bars represent standard deviations of the mean (*n* = 3–5 per group). Huh7 cells treated with cisplatin and doxorubicin. Representative microscopic images (**F**), immunofluorescence images (**G**), and results of qRT-PCR analysis (**H**) are shown. In **F** and **G**, cells were treated with drugs for four and two days, respectively. In **H**, Actb was used as an internal control, and error bars represent standard deviations of the mean (*n* = 3–9 per group). **I** Representative histograms showing the DNA content of Huh7-Fucci cells treated with DNA-damaging agents for four days. Data were obtained by flow cytometry, and cells in the G1 phase were analyzed. **J** Frequency of RPE1-Fucci cells in G1, S, and G2/M phases, and ploidy distribution of the cells in the G1 phase. Error bars represent standard deviations of the mean (*n* = 3–7 per group). **K**, **L** Chromosomal FISH analysis of drug-treated RPE1 cells. They were treated with each drug for 4 days. The distribution of chromosome numbers in the cells is shown in **K** (*n* = 100 cells/group). Representative fluorescent images are shown in **L**. Cisplatin was used at 2 μg/mL and 12 μg/mL for Huh7 and RPE1 cells, respectively. Doxorubicin was used at 100 ng/mL for Huh7 and RPE1 cells. NEBD nuclear envelop breakdown, CTR control, CDDP cisplatin, DXR doxorubicin, Chr chromosome. *p < 0.05, **p < 0.01. Student’s *t*-test. Scale bars, 100 μm in **A**, **F** and 10 μm in **G**, **L**.

We first examined alterations in cellular ploidy as the cells responded to DNA damage accumulation. The fluorescent ubiquitination-based cell cycle indicator (Fucci) system [15] was introduced into the human hyperdiploid hepatocellular cell line, Huh7, to assess cell cycle status and cellular ploidy (hereafter referred to as Huh7-Fucci cells, Fig. 1A) [16]. Flow cytometric analysis revealed a clear modal distribution of DNA content in G1 cells and replicated DNA content in cells in the S, G2, and M phases (Figs. 1B and S1). As previously reported [16], most Huh7-Fucci cells in G1 phase were diploid, whereas approximately 10% showed spontaneous polyploidy (Fig. 1C–E). Treatment with DNA-damaging agents, cisplatin and doxorubicin, at concentrations that entirely arrested cellular proliferation but allowed survival (Fig. S2) induced nuclear and cellular enlargement of Huh7-Fucci cells, the morphology of which was consistent with cellular senescence (Fig. 1F). No apparent reproliferation was observed after treatment with cisplatin and doxorubicin for four or more days and subsequent washout of the drugs for up to two weeks, suggesting that the proliferation of cells was irreversibly arrested as cellular senescence (Fig. S3). Upregulation of senescence markers, such as p16, p21, and IL6, was also confirmed (Fig. 1G, H).

Alterations in cellular ploidy during the progression of DNA damage-induced cell cycle arrest were investigated using flow cytometry. In cells treated with DNA-damaging agents, the proportion of cells in the S phase increased transiently as early as 2 days after drug treatment, and the proportion of cells in the G1 phase increased up to 7 days (Fig. 1D). DNA-damaging agents are presumed to induce transient cell cycle pauses in S-phase by disrupting DNA synthesis, followed by a gradual transition to G1-phase senescent cells. Although the proportion of polyploid cells among G1 cells increased during the process of senescence induction over time, approximately 30–50% of the cells remained diploid even after one week of drug treatment (Fig. 1E, I). p53 is thought to be an important regulator of ploidy [17], and Huh7 cells were transformed with p53 mutations, although the function of p53 in Huh7 cells was at least partially maintained (Fig. S4). Thus, we examined ploidy alterations during senescence induction elicited by DNA damage accumulation using the human non-transformed diploid cell line, RPE1, expressing Fucci (RPE1-Fucci), confirming polyploidization in a subset of senescent cells (Fig. 1J). These findings indicate that the process by which DNA damage accumulation leads to senescence induction is frequently accompanied by polyploidization; however, polyploidization is not essential for senescence induction.

To further examine polyploidization during senescence induction, we evaluated the increase in the nuclear and chromosomal numbers of senescent cells. Most (>90%) senescent RPE1 cells treated with cisplatin and doxorubicin were mononucleated, indicating that the polyploidization accompanied by DNA damage-induced senescence did not result from multinucleation. Moreover, fluorescence in situ hybridization (FISH) of chromosomes unexpectedly showed that the number of stained chromosomes was only slightly increased in senescent RPE1 cells compared to that in normal cells (Fig. 1K, L). Notably, the size and intensity of FISH signals were substantially higher in senescent cells than in control cells, and signals with two dots in pairs were occasionally observed (Fig. 1K, L). These findings suggest that senescent cells induced by DNA damage accumulation frequently become polyploid because of the lack of segregation of replicated chromosomes.

### The process of polyploidization results in the induction of genomic DNA damage

**Fig. 2:**
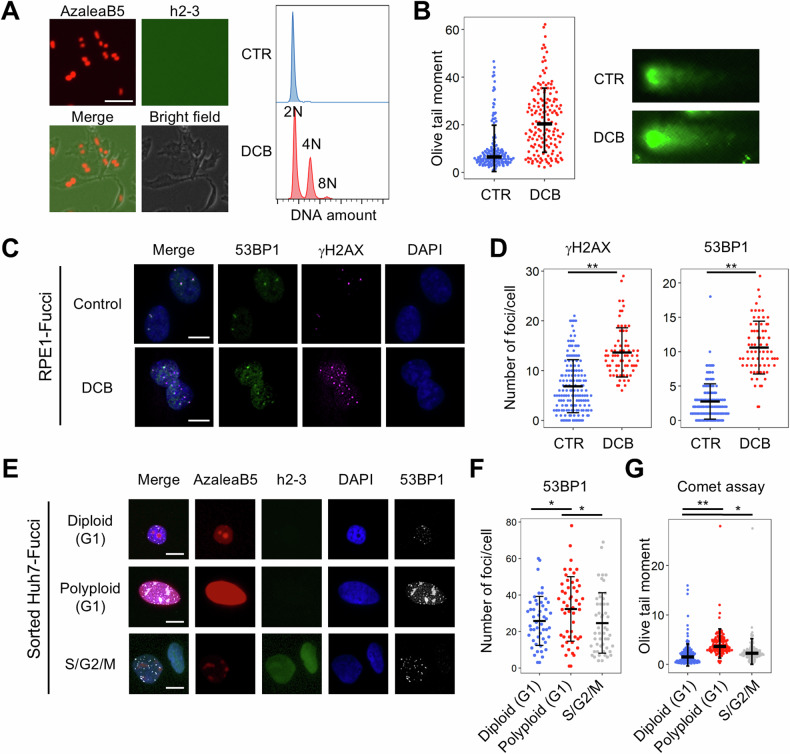
DNA damage accumulation in diploid and polyploid cells. **A**–**D** RPE1-Fucci cells were treated with DCB (10 μM) for 2 days. Microscopic images and histograms indicating the DNA content of G1 phase cells are shown in **A**. Neutral comet assay, representative immunofluorescence images, and the number of γH2AX- or 53BP1-positive foci per cell (*n* = 155 in control and 71 in DCB) are shown in **B**, **C**, and **D**, respectively. **E**–**G** Analysis of DNA damage accumulation in sorted Huh7-Fucci cells. G1 diploid, G1 polyploid, and S/G2/M cells were sorted using FACS from Huh7-Fucci cells cultured under normal conditions. Immunofluorescence images, the number of 53BP1-positive foci per cell (*n* = 50), and olive tail moments in neutral comet assay are shown in **E**, **F**, and **G**, respectively. In all dot plots, data were obtained from three or more independent experiments. Error bars represent the standard deviations of the mean. *p < 0.05, **p < 0.01. Student’s *t*-test. Scale bars, 50 μm in **A** and 10 μm in **C**, **E**.

Next, we examined whether polyploidization, a process of entire genome duplication, induces DNA damage. Diploid RPE1 cells were treated with dihydrocytochalasin B (DCB), an actin polymerization inhibitor, to induce polyploidization via incomplete cytokinesis (Fig. 2A). The neutral comet assay to detect double-stranded DNA (dsDNA) breaks showed that acutely polyploidized DCB-treated cells had more genomic dsDNA breaks than the control cells (Fig. 2B). Immunostaining for the markers of DNA damage foci 53BP1 and γH2AX also revealed that DCB-treated polyploid cells had more DNA damage (Fig. 2C, D). These findings suggest that the process of polyploidization promotes the accumulation of DNA damage, which is consistent with a recent report that polyploidization results in replication stress in the subsequent cell cycle [14].

We also examined the accumulation of DNA damage associated with polyploidization in the absence of drug treatment. Utilizing the features of Huh7 cells, a subset of which spontaneously become polyploid, we collected diploid and spontaneous polyploid Huh7-Fucci cells using fluorescence-activated cell sorting (FACS) and analyzed their genomic DNA damage. As expected, more genomic DNA damage was detected in spontaneous polyploid cells than in diploid cells in both immunostaining for DNA damage foci and the comet assay (Fig. 2E, F, G). Collectively, these results indicated that the process of polyploidization increases genomic DNA damage.

### Cells that are persistently polyploid are susceptible to accumulation of genomic DNA damage

**Fig. 3:**
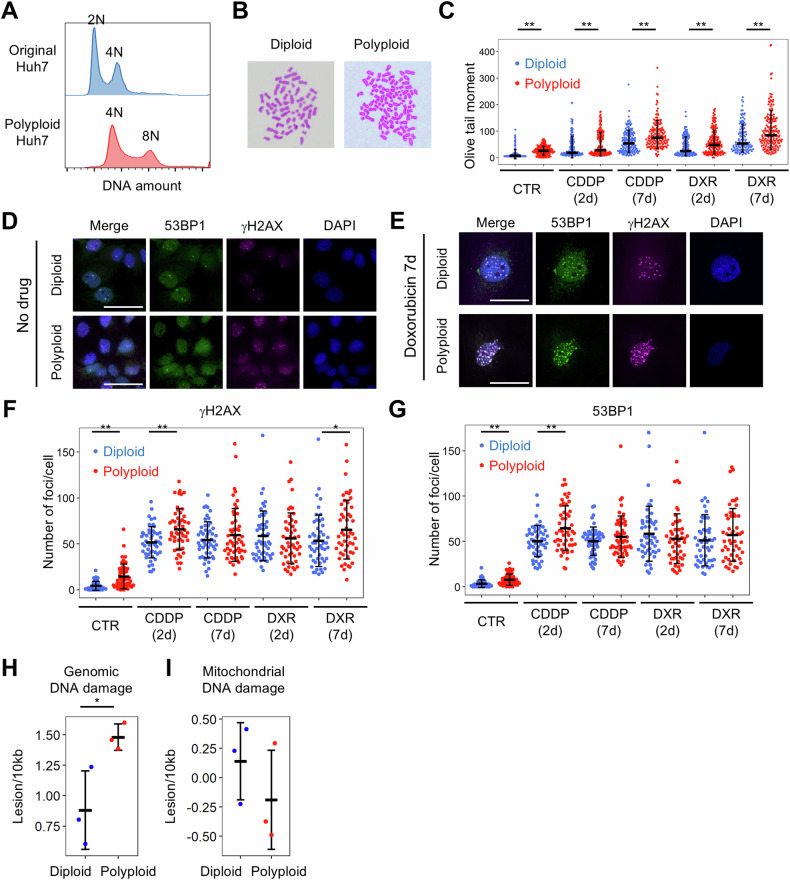
Comparison of DNA damage accumulation in diploid and stable polyploid Huh7 cells. **A** Representative histograms of the DNA content. **B** Metaphase chromosomes stained with Giemsa. **C** Olive tail moment in the neutral comet assay. *n* = 96–191 cells per group. **D**, **E** Immunofluorescence images of 53BP1 and γH2AX staining. **F**, **G** The number of γH2AX- or 53BP1-positive foci per cell. *n* = 50–52 cells per group. Diploid and stably polyploid Huh7-αTubulin-mScarlet cells were used in **C**–**G**. In **C**, **F**, and **G**, data were obtained from three or more independent experiments, and error bars represent the standard deviations of the mean. The number of genomic (**H**) and mitochondrial (**I**) DNA damage lesions evaluated by LORD-Q. Three independent diploid and stable polyploid cell lines established from Huh7 cells were analyzed in **H**, **I**. Each dot represents one cell line in **H** and **I**, and the variation in each cell line is shown in Fig. S5. Each cell line was analyzed in three independent experiments. The cells were treated with or without cisplatin and doxorubicin for a specified duration. CDDP cisplatin, DXR doxorubicin. *p < 0.05, **p < 0.01. Student’s *t*-test. Scale bar, 50 μm.

We further investigated whether persistent polyploidy was associated with DNA damage accumulation. As RPE1 cells polyploidized by DCB treatment can enter the subsequent cell cycle but cannot proliferate continuously [18], we could not establish stable polyploid RPE1 cells even after collecting polyploid G1 cells using FACS. In contrast, some cell lines that persistently exhibited polyploid were spontaneously obtained from Huh7 cells during the development of the novel stable cell lines (Fig. 3A). Giemsa staining of metaphase chromosomes confirmed that the established polypoid Huh7 cells grew continuously as polyploid cells (Figs. 3B and S5). The accumulation of dsDNA damage was compared in stable polyploid and diploid Huh7 cells using the comet assay, which revealed significantly more dsDNA damage in the polyploid cells (Fig. 3C). Immunostaining also revealed significantly more DNA damage foci in stable polyploid cells than in diploid cells (Fig. 3D, E, and F).

To investigate whether cells with persistent polyploidy are susceptible to the accumulation of extrinsic DNA damage as well as intrinsic DNA damage, diploid and stable polyploid Huh7 cells were treated with the DNA-damaging agents, cisplatin and doxorubicin. The comet assay consistently showed that the accumulation of dsDNA damage was significantly accelerated by drug treatment in polyploid Huh7 cells than that in diploid cells (Fig. 3C). Immunostaining for DNA damage foci supported this finding as well, although statistically significant differences were observed only under certain conditions (Fig. 3E–G). This may be because immunostaining for DNA damage foci indirectly detects genomic DNA damage, and the DNA damage response in polyploid cells could be somewhat alleviated, as discussed later. To confirm the enhanced accumulation of DNA damage in persistently polyploid cell lines, we evaluated genomic and mitochondrial DNA damage using a quantitative PCR-based evaluation system [19]. Notably, polyploid Huh7 cells showed significantly more damage to genomic DNA than diploid cells, with approximately twice the number of DNA lesions per unit base length (Figs. 3H, S6). This suggests that the increase in genomic DNA damage in stable polyploid cells was more than the increase in genomic DNA ploidy, compared to diploid cells. In contrast, no significant difference was observed in mitochondrial DNA damage per genomic ploidy (Figs. 3I, S6). Taken together, these results suggest that cells with persistent polyploidy constantly accumulate genomic DNA damage, but proliferate robustly, accepting more genomic damage than the diploid cells. The accumulation of DNA damage in polyploid cells is further enhanced by extrinsic DNA damage stress.

### Polyploid cells are tolerant to genomic DNA damage

**Fig. 4:**
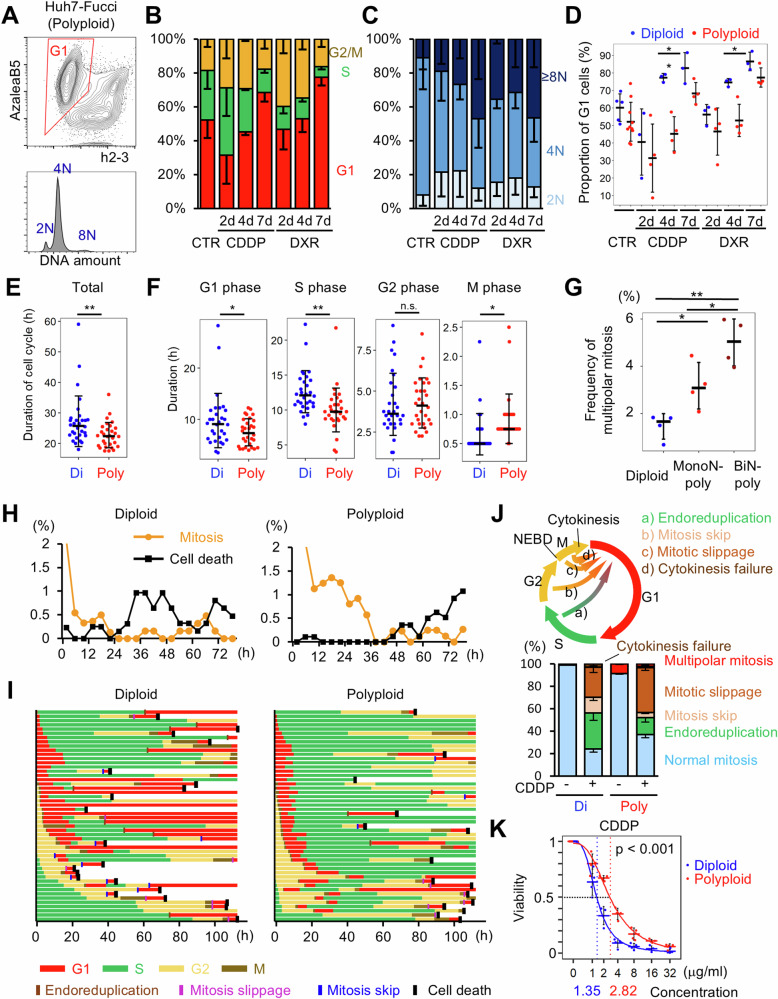
Time-lapse comparison of cell cycle progression between diploid and stably polyploid cells. **A** FACS plot of stable polyploid Huh7-Fucci cells without drug treatment. The lower panel shows the ploidy histogram of the G1 cells. **B**, **C** Frequencies of G1, S, and G2/M phases and the ploidy distribution of G1 cells. Stably polyploid Huh7-Fucci cells were treated with or without cisplatin and doxorubicin for a specified duration. Error bars represent standard deviations of the mean (*n* = 3–10 per group). **D** Comparison of the proportion of G1 cells between diploid and stable polyploid Huh7-Fucci cells (*n* = 3–10 per group). **E**, **F** Cell cycle duration. The total length of one cell cycle (**E**) and duration of each phase (**F**) are shown (*n* = 30 per group). **G** Frequency of multipolar mitosis without drug treatment (*n* = 4 per group). **H** Frequency of mitosis and cell death. **I** Cell cycle progression. In **H** and **I**, 50 cells per group were analyzed, and the x-axis indicates hours after the initiation of cisplatin treatment. **J** Frequency of abnormal cell cycles and their schemes. Cell divisions of Huh7-Fucci cells were evaluated for up to 114 hours after cisplatin administration (*n* = 200 in each group). Statistical results between diploid and polyploid cells are shown in Table S1. **K** Dose-response curve at 4 days after administration of cisplatin (*n* = 3 per group). The significance test for median lethal concentrations was performed using the bootstrap method. Panels **A**–**D** and **E**–**K** were obtained using FACS and microscopy, respectively. In all dot plots, error bars represent the standard deviations of the mean. CTR control, CDDP cisplatin, DXR doxorubicin, Di diploid, Poly polyploid, MonoN-poly Mononuclear polyploid, BiN-poly binuclear polyploid, NEBD nuclear envelop breakdown. *p < 0.05, **p < 0.01. Student’s *t*-test.

The robust proliferation of polyploid cells (Fig. S7A), despite harboring considerable DNA damage, suggests that polyploid cells possess a high level of tolerance for genomic damage accumulation. To evaluate ploidy-related differences in the susceptibility to genomic DNA damage, cell cycle alterations after treatment with DNA-damaging agents were compared between diploid and stable polyploid Huh7-Fucci cells (Fig. 4A). As observed in diploid Huh7-Fucci cells (Fig. 1D), flow cytometric analysis showed that treatment with cisplatin and doxorubicin transiently increased the proportion of cells in the S phase and subsequently increased the G1 proportion in polyploid Huh7-Fucci cells (Fig. 4B). Enhanced polyploidization was also observed even in stable polyploid cells (Fig. 4C). Notably, however, the proportion of G1-arrested cells was significantly lower among polyploid Huh7-Fucci cells than that in diploid cells (Fig. 4D). This suggests that the cell cycle arrest induced by DNA damage accumulation is delayed in polyploid cells, implying the mitigation of cellular stress by polyploidy.

To monitor the impact of ploidy on the cellular response to DNA damage in more detail, we performed time-lapse imaging of diploid and stable polyploid Huh7-Fucci cells. Under physiological conditions, the cell cycle duration of the stable polyploid cells was significantly shorter than that of the diploid cells (Fig. 4E). In particular, polyploid cells exhibited shorter G1 and S phases than diploids, confirming that polyploid cells proliferated robustly regardless of the accumulation of genomic DNA damage (Fig. 4F). In contrast, the duration of M phase in polyploid cells was significantly longer than that in diploid cells (Fig. 4F). Multipolar mitosis was significantly more frequent in polyploid cells than in diploid cells, suggesting that the difficulty in chromosome segregation due to polyploidy resulted in a longer M phase duration in polyploid cells (Fig. 4G, Supplementary movies).

Under cisplatin-treated conditions, mitosis, indicated by nuclear envelope breakdown, almost disappeared within 6 hours post the initiation of drug treatment (HPT) and cell death started to occur at approximately 24 HPT in diploid Huh7-Fucci cells (Fig. 4H). Notably, in marked contrast, stable polyploid cells underwent mitosis until 38 HPT and started to die at approximately 48 HPT (Fig. 4I). Moreover, while cell cycle progression entering the S phase was almost abolished by 24 HPT in diploid cells, stable polyploid cells successfully progressed to the subsequent cell cycle, even at approximately 40 HPT (Fig. 4I). These findings suggested that the cellular damage caused by cisplatin was significantly alleviated in cells with persistent polyploidy.

Cell cycle progression following cisplatin treatment can be categorized into five types: endoreduplication, mitosis skip, mitotic slippage, cytokinesis failure, and complete mitosis (Fig. 4J). Among these, endoreduplication (a direct transition from the h2-3^+^AzaleaB5^-^ S phase to the h2-3^-^AzaleaB5^+^ G1 phase) and mitosis skip (the transition from the h2-3^+^AzaleaB5^+^ G2 phase to the G1 phase without nuclear envelope breakdown), where cells do not enter the M phase, were significantly more common in diploid cells compared to polyploid cells, suggesting enhanced G2 checkpoint activation in diploid cells (Fig. 4J, Table S1). In contrast, polyploid cells underwent complete mitosis initially after cisplatin treatment at a significantly higher frequency compared to diploid cells, further supporting the enhanced tolerance to genotoxic damage by polyploidy (Fig. 4J, Table S1). Importantly, all four types of abnormal cell-cycle progression theoretically lead to polyploidization. Some cells that underwent endoreduplication or mitosis skip survived for more than 24 hours as G1-arrested cells, indicating that senescence with polyploidization was induced in these cells (Fig. S8, Table S2). In contrast, most of the cells that underwent mitotic slippage died. Some cells were arrested in the G1 phase after complete mitosis, giving rise to diploid senescent cells.

Furthermore, to confirm that cellular damage caused by genomic injury is mitigated in polyploid cells, we compared the survival rates of diploid and polyploid cells in response to various concentrations of cisplatin and doxorubicin. Notably, polyploid cells exhibited significantly reduced drug-induced cell death in response to both cisplatin and doxorubicin (Figs. 4K, S7B, and S7C). Collectively, these findings demonstrated that the consequences of genomic DNA damage, including cell cycle arrest and cell death, were alleviated in polyploid cells.

### Secretory phenotype associated with DNA damage-induced senescence is mitigated in senescent cells with polyploidization

**Fig. 5:**
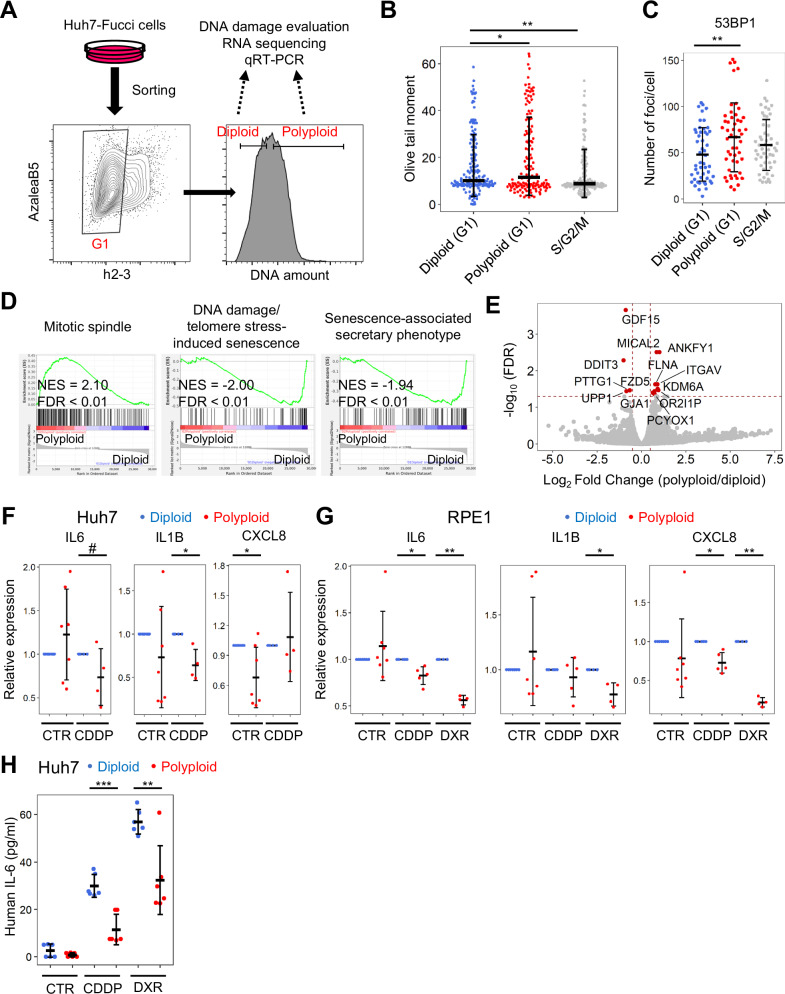
Transcriptome comparison between senescence induction with and without polyploidization. **A** Experimental scheme. Cellular senescence was induced by administering cisplatin to Huh7 cells for 4 days. **B** Olive tail moment in the neutral comet assay (*n* = 107–186 per group). **C** The number of 53BP1-positive foci per cell (*n* = 50–51 per group). **D**, **E** Gene set enrichment analysis (**D**) and volcano plot (**E**) comparing the transcriptomes in senescent cells with and without polyploidization. **F**, **G** Quantitative RT-PCR analysis of the sorted Huh7-Fucci (**F**) and RPE1-Fucci (**G**) cells (*n* = 4–7 per group). **H** IL-6 concentration in the cultured medium. The same number of diploid and polyploid Huh-Fucci cells were seeded and treated with cisplatin and doxorubicin. The culture medium was collected on day 4 and analyzed by ELISA (*n* = 6 per group). In all dot plots, error bars represent the standard deviations of the mean. FDR False discovery rate, NES Normalized enrichment score, CTR control, CDDP cisplatin. ^#^*p* = 0.10, *p < 0.05, **p < 0.01. Student’s *t*-test.

We further examined whether polyploidization alleviated the impact of DNA damage in senescent cells. Diploid Huh7-Fucci cells were treated with cisplatin, and diploid and polyploid cells arrested in the G1 phase were collected using FACS (Fig. 5A). The comet assay of the sorted cells showed that senescent cells with polyploidization had more genomic DNA damage than those without polyploidization (Fig. 5B). Immunostaining for 53BP1 revealed more DNA damage foci in the polyploid senescent cells than that in the diploid senescent cells (Fig. 5C). These results suggested that senescence induction accompanied by polyploidization involves more genomic DNA damage than senescence induction without polyploidization.

Next, we compared the expression profiles of senescent cells with and without polyploidization by analyzing the transcriptomes of the sorted cells. Gene set enrichment analysis (GSEA) demonstrated that like spontaneous polyploidization [16], genes related to mitotic spindles were upregulated in polyploid senescent cells, suggesting their association with polyploidization (Fig. 5D, Table S3). Notably, gene sets related to DNA damage-induced senescence and senescence-associated secretory phenotype (SASP) were significantly downregulated in senescent cells with polyploidization (Fig. 5D, Table S3). The suppression of secretory phenotypes related to DNA damage-induced senescence in polyploid cells was further suggested by the observation that growth differentiation factor-15 (GDF15), a stress-responsive cytokine, and DNA damage-inducible transcript 3 (DDIT3) were most significantly downregulated in senescent cells with polyploidization (Fig. 5E, Table S4).

To examine whether the SASP signature was alleviated in senescent cells with polyploidization, the expression of representative SASP cytokines [20], IL6, IL1β, and CXCL8, was evaluated in Huh7-Fucci and RPE1-Fucci cells treated with DNA-damaging agents. Quantitative RT-PCR of the sorted cells showed that senescent cells with polyploidization weakly expressed SASP cytokines compared to diploid senescent cells in both Huh7-Fucci cells and RPE1-Fucci cells (Fig. 5F, G). Furthermore, after treating equal numbers of diploid and polyploid Huh7-Fucci cells with CDDP for four days, significantly less IL6 was detected in the medium of polyploid cells compared to diploid cells by ELISA (Fig. 5H). Together with the observation that polyploid senescent cells harbored more DNA damage (Fig. 5B, C), these results suggest that polyploidization alleviates the impact of DNA damage and suppresses the secretory phenotype in cellular senescence.

### Polyploid hepatocytes are susceptible to the accumulation of genomic DNA damage in vivo

**Fig. 6:**
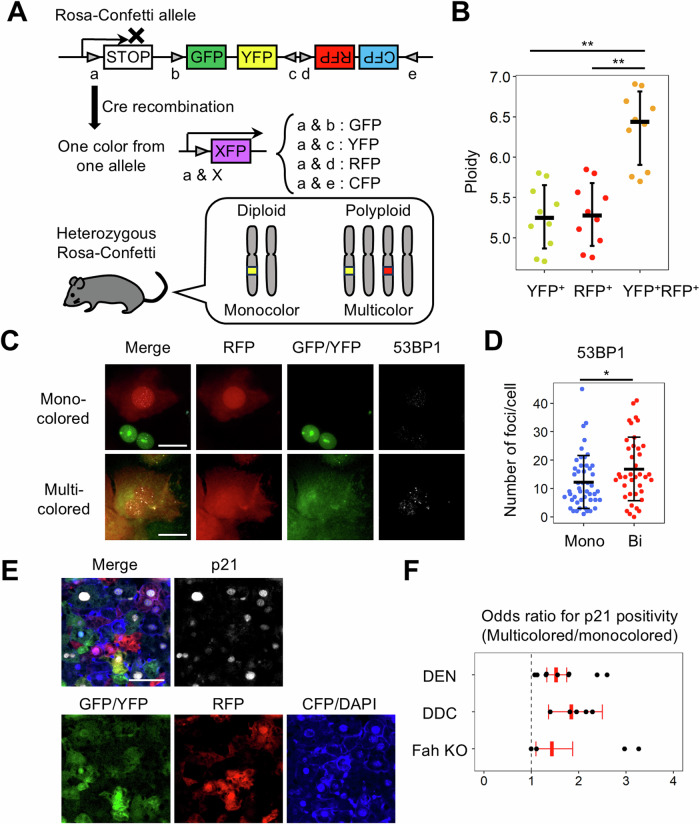
Implications of polyploidy in hepatocytes in vivo. **A** Scheme of genetic labeling in heterozygous Rosa-Confetti mice. Arrowheads indicate loxP sites. **B** Ploidy of mono- and multicolored hepatocytes. Hepatocytes were collected from heterozygous Rosa-Confetti mice 2 weeks after Cre recombination and examined by flow cytometry (*n* = 10 per group). **C** Immunofluorescence images of 53BP1 staining. Scale bar, 25 μm. **D** The number of 53BP1-positive foci per cell. Monoclored and bicolored hepatocytes were collected from the DEN-treated liver and examined by immunostaining (*n* = 48, and 39, respectively). In **B** and **D**, error bars represent the standard deviations of the mean. **E** Immunofluorescence images of p21. Scale bar, 50 μm. **F** Odds ratio for p21 positivity in multicolored hepatocytes compared with that in monocolored hepatocytes. Mouse livers injured with DEN administration (*n* = 9), DDC diet feeding (*n* = 5), and Fah deficiency (*n* = 4) were analyzed. Error bars indicate 95% confidence intervals. KO knockout. *p < 0.05, **p < 0.01. Student’s *t*-test.

Finally, we examined whether polyploid hepatocytes in the liver accumulated genomic damage. As cellular ploidy can be altered by liver injury, we utilized an in vivo lineage-tracing system that enables the tracking of polyploid cells over time as multicolored cells [21, 22]. In heterozygous Rosa-Confetti mice, all multicolored hepatocytes were polyploid [21, 22] and exhibited significantly higher ploidy than monocolored hepatocytes (Fig. 6A, B). Heterozygous Rosa-Confetti mice, whose hepatocytes were selectively labeled by an adeno-associated virus expressing Cre under the control of a hepatocyte-specific promoter, were treated with a liver genotoxic agent, diethylnitrosamine (DEN), and DNA damage foci in hepatocytes were examined by immunostaining. Multicolored hepatocytes had significantly more 53BP1-positive foci in the nucleus than those in monocolored hepatocytes, indicating that polyploidy was associated with enhanced DNA damage accumulation in vivo (Fig. 6C, D).

To further examine the impact of ploidy on the response to hepatocytic injury, various types of chronic liver injury were induced in heterozygous Rosa-Confetti mice. Histological analysis showed that hepatocyte death was minimal in the damaged livers in all the liver injury models examined (Fig. S9). Notably, the odds ratios for p21 positivity in multicolored hepatocytes compared to monocolored hepatocytes were greater than 1 in all liver injury models, indicating that multicolored hepatocytes were positive for p21 at a significantly higher frequency than monocolored hepatocytes (Fig. 6E, F). The p21-positive hepatocytes were negative for Ki-67 and assumed to be cell cycle-arrested cells induced by cellular damage. Considering that p21 is critically involved in the repair of DNA damage and the inhibition of apoptosis in the context-specific p53 responses to DNA damage [23], hepatocytes with high ploidy appeared to potentially persist in the damaged liver while being susceptible to DNA damage.

## Discussion

In the present study, we demonstrated a close positive relationship between polyploidy and accumulation of genomic damage. As excessive DNA damage can result in abnormal cell cycle progression, leading to polyploidization [24, 25], it is to be expected that polyploid cells harbor damaged genomic DNA. In addition, the polyploidization process has recently been shown to enhance replication stress in the subsequent cell cycle, eliciting DNA damage [14], which is consistent with our findings in DCB-induced and spontaneous polyploid cells. Importantly, we also demonstrated that cells proliferating as stable polyploid cells sustained an elevated risk of genomic DNA damage and harbored more dsDNA breaks and DNA damage foci than diploid cells, indicating that the state of polyploidy, as well as the polyploidization process, leads to genome instability. Notably, compared to diploid cells, polyploid cells exhibit a shorter duration of the G1 phase, which could impose enhanced replication stress [26, 27]. Moreover, as suggested by the prolonged M phase and frequent multipolar mitosis, stable polyploid cells are prone to chromosome segregation errors, which can also elicit DNA damage [28]. Polyploid cells may be more genomically unstable during proliferation than diploid cells, leading to constant intrinsic accumulation of genome damage in stable polyploid cells.

Although polyploid cells harbor genomic damage that is more than the increase in ploidy compared to diploid cells (Fig. 3H), the likelihood that essential genes are damaged in all duplicated alleles (e.g. 4-hits in tetraploid cells) is expected to be much lower than the possibility of a 2-hit occurrence in diploid cells. This can explain the alleviated cellular responses in polyploid cells, such as delayed cell death, following treatment with DNA-damaging agents. The buffering of genomic damage by polyploidy would be highly advantageous, especially in the liver [29], which is at high risk of exposure to genotoxic molecules. Hepatocytes are a representative polyploid cell type, and their physiological and enhanced polyploidization in injured livers would protect them from lethal cellular damage [5]. However, polyploid cells that thrive despite harboring substantial DNA damage can be the origin of carcinogenesis as reservoir of genomic abnormalities. Indeed, genome duplication during carcinogenesis is estimated to occur following genetic alterations in key cancer-related genes in some cancers [10]. Polyploidization may enhance carcinogenesis by promoting conditions that accommodate genomic abnormalities, in addition to leading to chromosomal instability. Moreover, polyploidy-enhanced tolerance to genomic damage accumulation can result in the acquisition of resistance to chemotherapeutic agents such as cisplatin. While enhanced adaptability to genomic damage due to polyploidy can be advantageous for enduring tissue injury, it may also give rise to cellular evolution or robustness in the context of cancers.

Given that polyploid cells accumulate many genomic abnormalities, the proliferation of potential cancer-initiating cells must be prevented. Cellular senescence, an irreversible cell cycle arrest induced by activation of the DNA damage response pathway, acts as an important barrier to cancer development by inhibiting the proliferation of abnormal cells. The processes of polyploidization and the induction of cellular senescence are closely related, as they are often observed simultaneously [30, 31]. Importantly, we showed that the induction of cellular senescence is not necessarily accompanied by polyploidization, indicating that polyploidization is not an obligate cellular change that ensures cell cycle arrest. Consistently, while polyploidization has been considered to induce the arrest of cell division in the past [32], normal polyploid hepatocytes can proliferate extensively to regenerate the liver [21]. On the other hand, the implication of polyploidy in cell cycle arrest in PGCCs is posited by the observation that PGCCs exhibiting senescent traits, induced by genotoxic stress caused by anti-cancer therapy, reinitiate their proliferation through de-polyploidization [12, 33]. Given these facts and the attenuated expression of inflammatory SASP factors in polyploid senescent cells, polyploidy may be a unique feature for classifying heterogeneous senescent cells. The characteristics of polyploid senescent cells, including PGCCs, compared to those of diploid senescent cells need to be further explored.

In conclusion, we demonstrated that polyploidy acts as a reservoir of genomic damage while buffering its impact. Physiological and disease-related polyploidization can protect cells against damage and may be involved in responses to tissue injury and cancer development. Since duplication of the whole genome is undoubtedly a critical change in cells, further investigation is required to understand its implications for cellular behavior and pathogenesis.

## Materials and methods

### Cell culture

The human hepatoma cell line Huh7 and retinal pigment epithelium cell line RPE1 were obtained from the Japanese Collection of Research Bioresources (Osaka, Japan) and Lonza Inc. (Basel, Switzerland), respectively. Cells were maintained in Dulbecco’s modified Eagle’s medium (Nakarai Tesque, Kyoto, Japan) supplemented with 10% fetal bovine serum (Biowest, Nuaillé, France). No mycoplasma contamination was detected in any of the cell lines. Huh7-Fucci and RPE1-Fucci cells were described previously [16]. Huh7 cells expressing the α-Tublin-mScarlet fusion gene or the histone H2B-mNeonGreen fusion gene were established using the Sleeping Beauty transposon system in a manner similar to a previously reported method [16]. A sleeping-beauty transposon plasmid encoding the α-Tublin-mScarlet fusion gene was constructed by subcloning the α-Tublin-mScarlet fusion gene amplified from pmScarlet_alphaTubulin_C1 (Addgene plasmid # 85045) [34] using PCR into pSBbi-RP (Addgene plasmid # 60513) [35] after deleting the *RFP* gene in pSBbi-RP. The histone H2B-mNeonGreen fusion gene was created by modifying mEos2-H2B-6 (Addgene plasmid # 57384) [36] and 3xnls-mNeonGreen (Addgene plasmid # 98875) [37] and was subcloned into a sleeping-beauty transposon plasmid harboring a puromycin selection cassette. Huh7 cells were transfected with the plasmids described above and pCMV(CAT)T7-SB100 (Addgene plasmid # 34879) [38] encoding the SB100X transposase. Cells with stable DNA integration were selected using puromycin for over a month. Overall, six batches of stable cell lines were established independently, including the diploid and stable polyploid cell lines, Huh7-Fucci, Huh7-αTubulin/mScarlet, and Huh7-H2B/mNeonGreen. These six cell lines, along with the parental Huh7 cells, were used in this study. The cells were treated with cisplatin (2 μg/mL for Huh7 and 12 μg/mL for RPE1; FUJIFILM Wako Pure Chemical, Osaka, Japan), doxorubicin (100 ng/mL for Huh7 and RPE1; FUJIFILM Wako Pure Chemical), DCB (10 μM for RPE1; Merck, Darmstadt, Germany), and pifithrin α (20 μM for Huh7; MedChemExpress, Monmouth Junction, NJ), unless otherwise specified.

### Immunostaining and histology

Cultured cells and frozen mouse tissues were fixed in 4% paraformaldehyde and subjected to immunofluorescence staining. Immunofluorescence was performed with primary and secondary antibodies including mouse anti-phospho-Histone H2A.X (Ser139) (1:1000, Sigma-Aldrich, cat#: 05-636), rabbit anti-53BP1 (1:600, Santa cruz, Dallas, TX, cat#: sc-22760), rabbit anti-p21 (1:200, Abcam, Cambridge, UK, cat#: ab188224), rabbit anti-Ki67 (1:200, Abcam, cat#: ab15580), Alexa Fluor Plus 488-conjugated anti-rabbit (1:200, Thermo Fisher Scientific, cat#: A32790), Alexa Fluor 594-conjugated anti-mouse (1:200, Thermo Fisher Scientific, cat#: A21203) and Alexa Fluor 647-conjugated anti-rabbit (1:200, Thermo Fisher Scientific, cat#: A31573). The nuclei were counterstained with DAPI (F10347; Thermo Fisher Scientific, Waltham, MA, USA). Fluorescence images were analyzed using a fluorescence microscope (BZ-X710; Keyence, Osaka, Japan) and BZ-X analyzer software (Keyence).

### Flow cytometry and fluorescence-activated cell sorting (FACS)

Cultured cells were harvested from the dishes using TrypLE (Thermo Fisher Scientific). Mouse primary hepatocytes were isolated by digesting the liver using a two-step collagenase perfusion method as described previously [21]. The collected cells were suspended in phosphate buffer solution containing 3% fetal bovine serum. Cells were incubated with 5 μM Vibrant DyeCycle Violet stain (Thermo Fisher Scientific) for 30 min at 37 °C to detect ploidy. Dead cells were excluded by staining with propidium iodide (Sigma-Aldrich). Stained cells were analyzed using Attune NxT (Thermo Fisher Scientific) or sorted using a BD FACSAria III (Becton Dickinson, NJ, USA) or BD FACSymphony S6 (Becton Dickinson). In the analysis of drug-treated cells, where the modal distribution of ploidy was disrupted, untreated control samples were analyzed in parallel as references to accurately distinguish ploidy levels. In the analysis of Rosa-Confetti hepatocytes, only the expression of YFP and RFP was considered among the four fluorescent proteins encoded in the Rosa-Confetti allele because the signal of CFP is too weak to detect and GFP is rarely expressed in hepatocytes. Sorted cells were collected in RNAprotect Cell Reagent (Qiagen, Venlo, Netherlands) and stored in a deep freezer until processing. Flow cytometry data were processed using the FlowJo software (TreeStar).

### Quantitative reverse transcription polymerase chain reaction (qRT-PCR)

Total RNA was isolated from cells using TRIzol (Thermo Fisher Scientific) or an RNeasy Micro Kit (Qiagen), according to the manufacturer’s protocol. Complementary DNA was synthesized using the PrimeScript RT Reagent Kit with gDNA Eraser (Takara Bio Inc., Shiga, Japan). The qRT-PCR was performed on a Thermal Cycler Dice Real-Time System III (Takara Bio) using TB Green Premix Ex Taq II (RR820A; Takara Bio). The expression level of each gene was calculated relative to the expression levels of the housekeeping genes β-actin, GAPDH and PSMB6. Primers used are listed in Table S5.

### Chromosome FISH

Chromosome FISH was performed using probes specific to the alpha satellite regions of chromosomes 2 (#LPE002G) and 3 (#LPE003R) according to the manufacturer’s instructions (Oxford Gene Technology, Oxford, United Kingdom). Nuclei were counterstained with DAPI. The FISH slides were examined at 100× magnification using a fluorescence microscope (BZ-X710, Keyence).

### Giemsa staining

After the treatment with colcemid (FUJIFILM Wako Pure Chemical) at 0.1 μg/mL for 30 minutes, cells were harvested and collected by centrifugation. The collected cells were then immersed in a hypotonic KCl solution (0.075 mol/L) for 20 minutes and fixed with a freshly prepared methanol/glacial acetic acid solution (3:1 v/v). The fixed cells were washed with a fixative three times and dropped onto microscope slides. After air-drying, the slides were stained with a 4% Giemsa staining solution (KaryoMax Giemsa Stain Solution in Gurr buffer; Thermo Fisher Scientific). Images were acquired using a DP-27 microscope (Olympus, Tokyo, Japan). Fifty metaphase chromosome spreads were counted for each cell line.

### Neutral comet assay

The cultured cells were harvested and resuspended in PBS to 2 × 10^5^ cells/mL. The cell suspension was mixed with 1% low-melting agarose (Lonza) at 3/4 ratio (v/v). The mixture was dropped onto slides precoated with low melting agarose gel, and slides were incubated with lysis solution (Trevigen, Gaithersburg, MD) for 30 minutes at 4 °C. Neutral electrophoresis was performed for 20 minutes at 30 volts in TBE buffer, and the slides were rinsed with 70% ethanol for 5 minutes and allowed to air-dry. DNA was stained with Midori Green Direct (Nippon Genetics, Tokyo, Japan) and images were acquired using a fluorescence microscope (BZ-X710, Keyence). The olive tail moment, defined as the percentage of DNA in the tail multiplied by the tail moment length, was quantified using CometScore 2.0 software. In all the experiments, the olive tail moment was calculated based on more than 100 comet tails.

### LORD-Q

The LORD-Q method was performed as described by Lehle et al. [19]. Briefly, total DNA was extracted from cells using the PureLink Genomic DNA Mini Kit and quantitative real-time PCR was performed on a Thermal Cycler Dice Real Time System III (Takara Bio) using the KAPA SYBR FAST qPCR kit (Roche, Basel, Switzerland). The DNA damage frequency was calculated based on the original LORD-Q method [19]. Primers used are listed in Table S6.

### Evaluation of mitochondrial number

The number of mitochondria was determined by real-time PCR on a Thermal Cycler Dice Real-Time System III (Takara Bio) using a Human Mitochondrial DNA Monitoring Primer Set (Takara Bio). The amount of mitochondrial DNA relative to that of genomic DNA was calculated according to the manufacturer’s protocol.

### Time-lapse imaging

For fluorescence time-lapse imaging, Huh7-Fucci cells were maintained in FluoroBrite DMEM Media (Thermo Fisher Scientific) supplemented with 10% fetal bovine serum, 1% penicillin/streptomycin, GlutaMAX (Thermo Fisher Scientific), and HEPES (Thermo Fisher Scientific) at 37 °C and 5% CO2. Images were acquired every 15 minutes for 114 hours using a fluorescence microscope (BZ-X 810, Keyence) and analyzed using the Fiji software and ZEN3.2 (blue edition) (ZEISS, Germany).

### RNA sequencing analysis

Total RNA was extracted from sorted cells using an RNeasy Micro Kit (Qiagen). Full-length cDNA was generated using the SMART-Seq HT Kit (Takara Bio), and an Illumina library was prepared using the Nextera DNA Library Preparation Kit (Illumina, San Diego, CA). Sequencing was conducted using an Illumina NovaSeq 6000 sequencer (Illumina) in the 100-base paired-end mode at the Genome Information Research Center, Research Institute for Microbial Diseases, Osaka University. Sequencing reads were mapped to the GRCh38 human reference genome (Ensembl release 100) using STAR (version 2.7.4a), and the reads per gene were counted using HTSeq (version 0.12.4). Differential gene expression analysis was performed using the edgeR software (version 3.30.3). Statistical significance was assessed using quasi-likelihood F-tests, and the *p*-values were corrected for the false discovery rate (FDR) using the Benjamini–Hochberg method at a threshold of 0.05. Gene set enrichment analysis was performed by the Broad Institute GSEA (version 4.2.0) using the gene list pre-ranked according to the singed fold change × ( − log10 FDR).

### Sandwich ELISA assay

Huh7 diploid and polyploid cells were seeded at 5.26 × 10^3^ cells/cm^2^ each, with or without DNA damaging agents (cisplatin 2 μg/ml or doxorubicin 100 ng/ml) and the culture supernatant was collected after 4 days. The concentration of IL-6 in the supernatant was determined using the ELISA MAX Deluxe Set Human IL-6 (BioLegend cat#: 430504) and the absorbance was measured for each sample using the SYNERGY HTX (Bio Tek). The concentration was calculated based on the 4 Parameter Logistic approximation curve using GainData (https://www.arigobio.com/elisa-calculator).

### Animal experiment

Rosa-Confetti mice [39] were obtained from The Jackson Laboratory. Fah^−^^/−^ mice on the C57BL/6 were provided by Dr. Markus Grompe (Oregon Health & Science University) and maintained on 8 mg/L 2-(2-nitro-4-trifluoromethylbenzoyl)-1,3-cyclo-hexanedione (NTBC; AmBeed, Arlington Heights, IL) in drinking water until the experiments. Heterozygous Rosa-Confetti mice were injected with AAV8-Ttr-Cre [40] at 8 or more weeks old, and liver injuries were induced in mice 2 weeks or more after AAV administration. The AAV-Ttr-Cre construct was provided by Dr. Holger Willenbring (University of California, San Francisco). DEN was administered to mice twice a week at a dose of 100 μL/25 g body weight for 2 weeks. Chow containing 0.1% (w/w) 3,5-diethoxycarbonyl-1,4-dihydrocollidine (DDC; Sigma-Aldrich) was fed to the mice for four weeks. To induce liver injury caused by Fah deficiency, Rosa-Confetti^+/−^;Fah^−^^/−^ mice were subjected to cyclic withdrawal of NTBC (10 days off, 4 days on). Mouse livers were harvested after a recovery period of more than one week in the DEN-induced and Fah-deficient liver injury models. The mice were maintained under temperature- and humidity-controlled conditions and exposed to a 12-h light–dark cycle. All the mouse experiments were approved by the Animal Research Committee of the Research Institute for Microbial Diseases, Osaka University.

### Statistical analysis

Statistical analyses were performed using the Mann–Whitney U test, Student’s *t*-test, chi-square test, or bootstrap method using R software (version 4.1.2, R Foundation for Statistical Computing) and Microsoft Office Excel (Microsoft). No statistical methods were used to predetermine sample sizes, but our sample sizes are similar to those reported in previous publications [14, 16, 26, 41].

## Supplementary information

Supplement Figure

Supplemental Tables

Supplementary movie 1

Supplementary movie 2

Supplementary movie 3

## Data Availability

The RNA sequencing data generated in this study have been deposited in the DDBJ Sequence Read Archive (https://www.ddbj.nig.ac.jp) under accession number DRA017341. The authors declare that all other data are available upon request.
