# Supplementary material for: Polyploidy mitigates the impact of DNA damage while simultaneously bearing its burden
Source: Cell Death Discov. 2024 Oct 13;10:436. doi: 10.1038/s41420-024-02206-w (PMC11471775; doi:10.1038/s41420-024-02206-w)
Supplement: Supplementary file 1 — Supplement Figure [file 41420_2024_2206_MOESM1_ESM.pdf]

Polyploidy mitigates the impact of DNA damage while simultaneously bearing its burden

Kazuki Hayashi, Kisara Horisaka, Yoshiyuki Harada, Yuta Ogawa,

Takako Yamashita, Taku Kitano, Masahiro Wakita, Takahito Fukusumi,

Hidenori Inohara, Eiji Hara, Tomonori Matsumoto

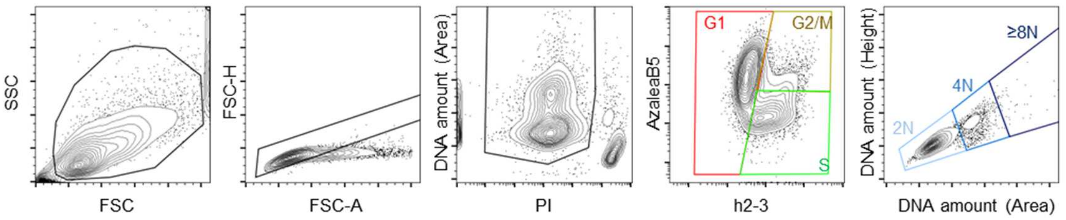

Figure S1. Gating strategy for flow cytometry analysis of Huh7-Fucci cells

FSC, forward scatter; SSC, side scatter.

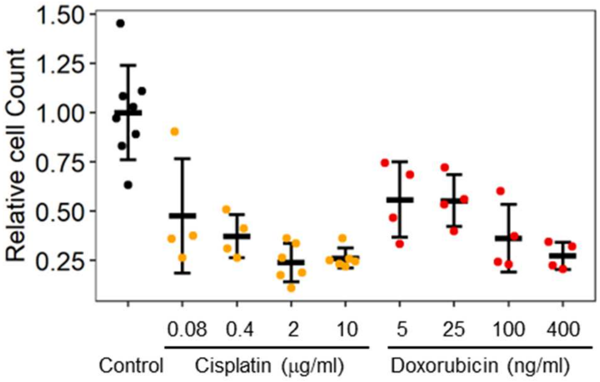

Figure S2. Suppression of cell proliferation by drugs

Huh7 cells were treated with cisplatin and doxorubicin at the indicated concentrations for three days. Relative cell numbers were calculated to standardize the average number of untreated control cells to 1 (n = 4–8 per group). Error bars represent standard deviations of the mean.

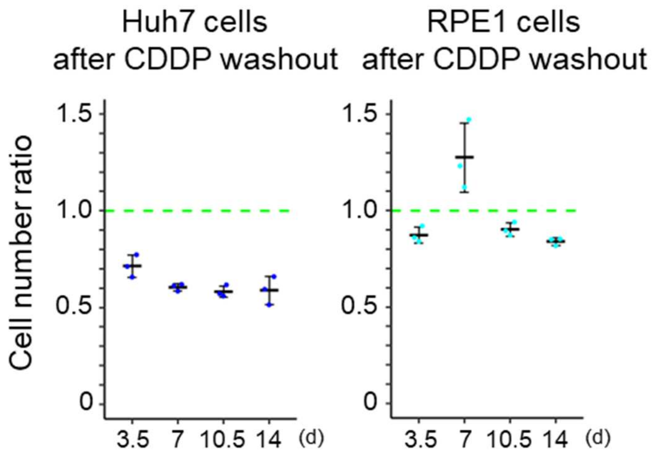

Figure S3. Cell number ratio after the drug washout

Huh7 and RPE1 cells were seeded at 1.58 × 10^4 cells/cm^2 and 7.89 × 10^3 cells/cm^2, respectively and treated with cisplatin (Huh7: 2μg/ml, RPE1: 12μg/ml) for four days, followed by drug washout for up to two weeks. The day the drug was washed out was designated as day 0. The cell number ratio was calculated by setting the number of cells at the previous observation (3.5 days prior) to 1 (n = 3 per group). Error bars represent standard deviations of the mean. At all observation points, no significant variation exceeding 1 was observed by Student's t-test.

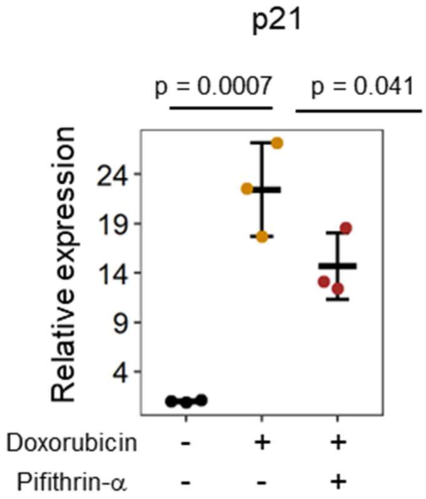

Figure S4. qRT-PCR analysis of Huh7 treated with doxorubicin and Pifithrin-α

Huh7 cells were treated with doxorubicin (100 ng/mL) and pifithrin α (20μM) for 2 days. ACTB was used as an internal control, and the relative expression levels of the *p21* gene normalized to those of the control cells are shown (n = 3 per group). Statistical significance was determined using two-tailed unpaired Student's t-test. Error bars indicate mean ± SD.

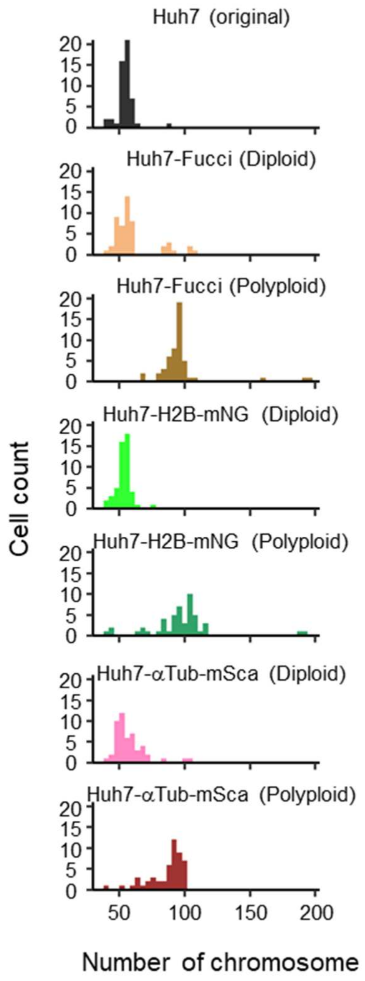

Figure S5. Chromosome numbers of diploid and stably polyploid Huh7 cells

Histograms of chromosome numbers per cell. The number of chromosomes stained with Giemsa was counted in approximately 50 cells per cell line (n = 49-51). Huh7-H2B-mNeonGreen, Huh7-H2B-mNG; Huh7-αTublin-mScarlet, Huh7-aTub-mSca.

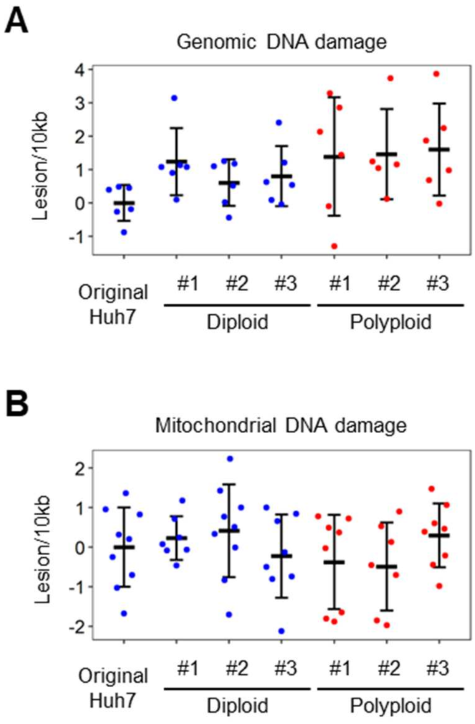

Figure S6. The amount of genomic and mitochondrial DNA damage lesion evaluated by LORD-Q

Genomic (A) and mitochondrial (B) DNA damage in three different diploid and stable polyploid cell lines derived from original Huh7 were assessed by the LORD-Q method. Results from six independent experiments are shown. The average of the six measurements for each cell line was considered the DNA damage level for that cell line, and these average values are shown in Figure 3H.

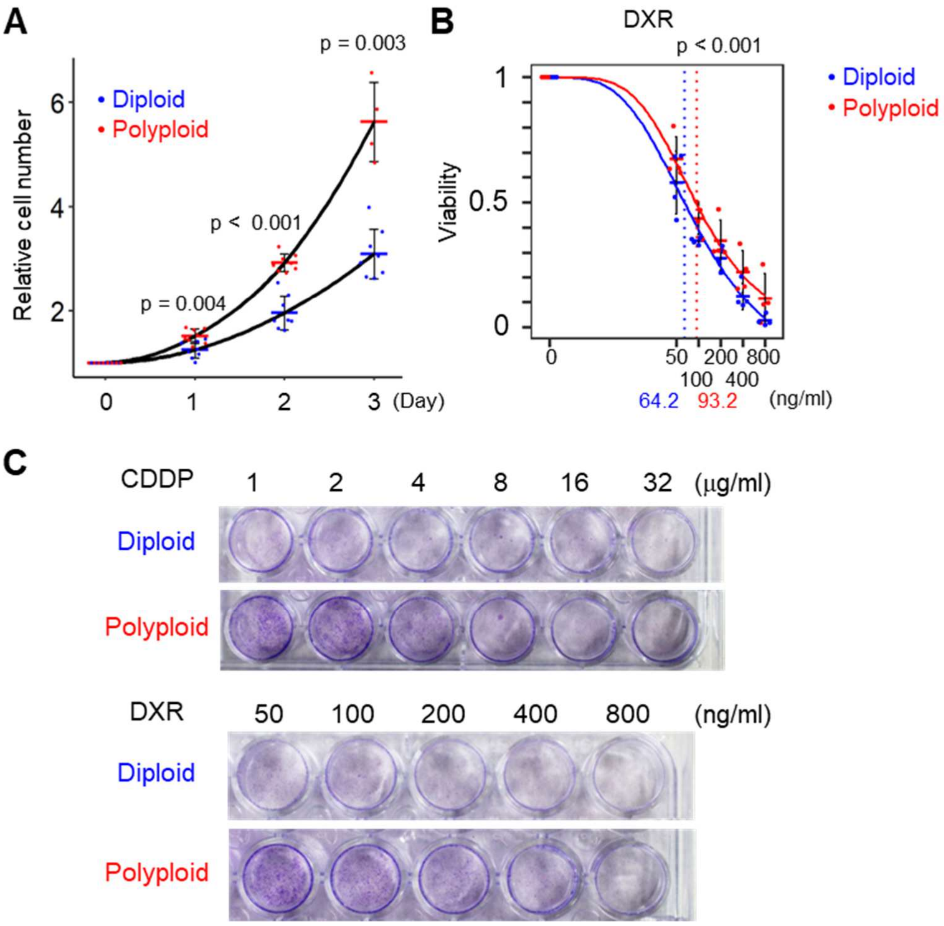

Figure S7. Dynamics of diploid and polyploid Huh7-Fucci cells with and without DNA-damaging agents

(A) Proliferation curves of diploid and polyploid Huh7-Fucci cells without drug treatment. (B) Dose-response curve to doxorubicin in diploid and polyploid Huh7-Fucci cells. The median lethal concentrations of diploid and polyploid cells are shown in blue and red, respectively. (C) Crystal violet staining. Diploid and polyploid Huh7-Fucci cells were seeded into each well at the same number (3×10^4 cells per well) on the first day and stained with crystal violet four days after the drug treatment. Statistical significance was determined using

a two-tailed unpaired Student’s t-test in (A). The significance test for median lethal concentrations was performed using the bootstrap method.

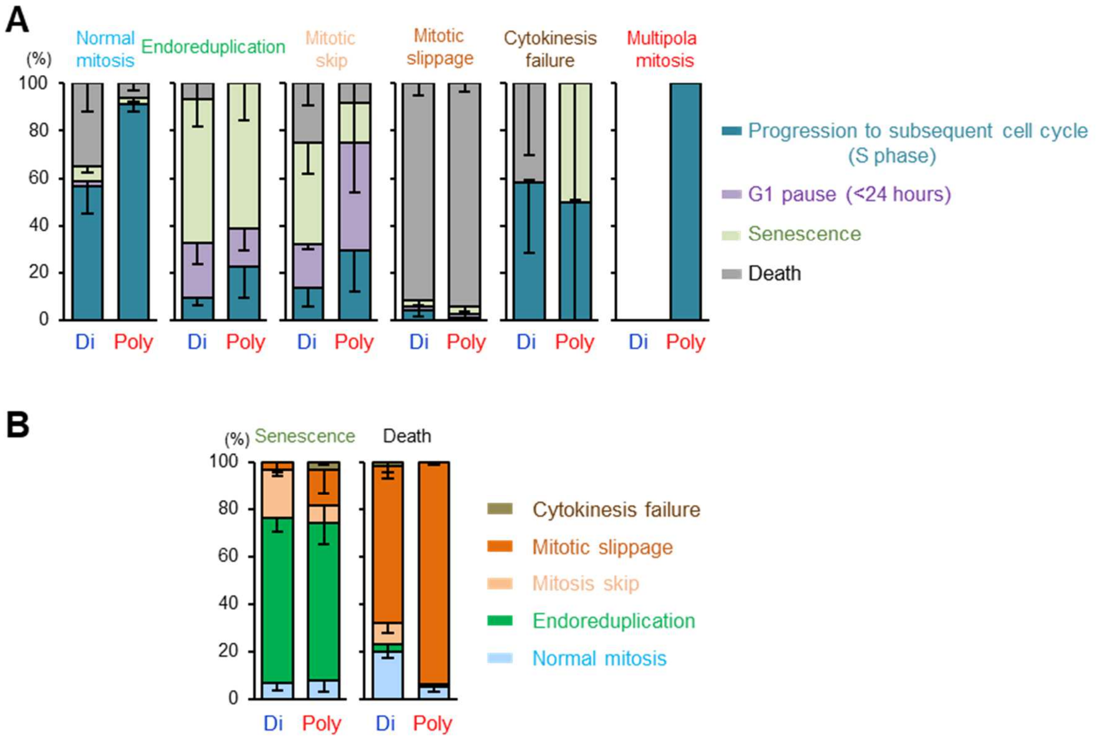

Figure S8. Relationship between types of cell cycles and cellular fate

The relationship between mitotic abnormalities in Huh7-Fucci cells and their subsequent fate was evaluated for up to 114 hours after cisplatin administration (n = 200 in each group). (A) Cellular fate in each type of cell cycles. (B) Types of cell cycles leading to cellular senescence and cell death. Statistical results between diploid and polyploid cells are shown in Table S2.

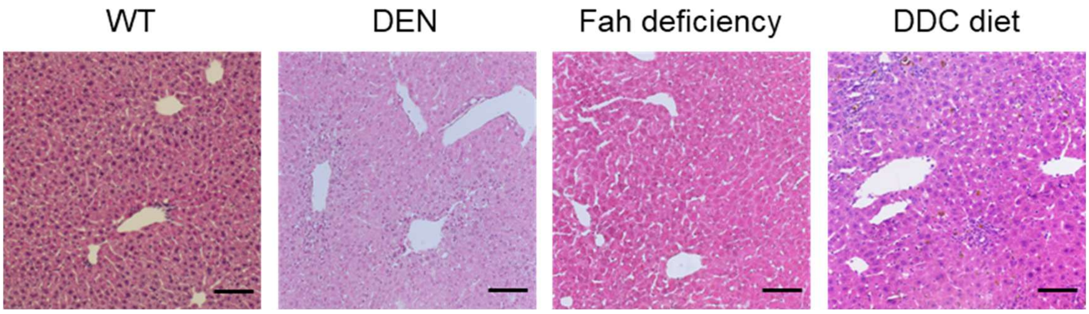

Figure S9. HE stains of mouse liver tissue

Representative HE-stain images of the liver of wild-type mice and mice with liver injuries.

WT, wild type. Scale bar, 100 μm.

Legends for Supplementary Tables and Supplementary Movies

Table S1. Statistical results of the data in Figure 4J

The p-values resulting from Student's t-tests between diploid and polyploid cells are shown.

Table S2. Statistical results of the data in Figure S7

The p-values resulting from Student's t-tests between diploid and polyploid cells are shown.

Table S3. Results of gene set enrichment analysis comparing senescent cells with and without polyploidization.

Gene sets with a false discovery rate q-value below 0.01 are listed.

Table S4. Differentially expressed genes between diploid and polyploid senescent cells

Table S5. Primers used for qRT-PCR analysis

Table S6. Primers used for LORD-Q assay

Supplementary movie 1. Normal mitosis in a diploid Huh7-Fucci cell

Supplementary movie 2. Multipolar mitosis in a mononuclear polyploid Huh7-Fucci cell

Supplementary movie 3. Multipolar mitosis in a binuclear polyploid Huh7-Fucci cell
